# TRF2 Recovers Ischemic Postconditioning Cardioprotection in Aged Myocardiocytes by Regulating CSNK2A2 Localization and FUNDC1 Dephosphorylation

**DOI:** 10.1111/acel.70669

**Published:** 2026-08-17

**Authors:** Xuan Zhang, Ji Ma, Ruichen Shu, Yuan Li, Ying Zheng, Yiqing Yin

**Affiliations:** ^1^ Key Laboratory of Cancer Prevention and Therapy, Department of Anesthesiology Tianjin Medical University Cancer Institute and Hospital, National Clinical Research Center for Cancer, Tianjin's Clinical Research Center for Cancer Tianjin China; ^2^ Department of Anesthesiology Tianjin Union Medical Center Nankai University Affiliated Hospital Tianjin China

**Keywords:** aged myocardiocytes, cGAS/STING pathway, ischemic postconditioning, mitophagy, TRF2

## Abstract

Ischemic postconditioning (I/Post), which is an effective intervention by activating endogenous cardioprotective pathways, recovers ischemia/reperfusion injury. However, this intervention is not as effective in older patients, and its mechanism needs to be further investigated. In this study, we found that myocardial telomeric repeat binding factor 2 (TRF2) protein expression in male aged mice (18 months of age) was lower than that in male adult mice (4 months of age). After ligation of the anterior descending branch of the heart to establish an in vivo model of ischemia/reperfusion injury, we found that TRF2 expression was further decreased after I/Post. To investigate the role of TRF2 in cardioprotection in I/Post in the senescent heart, we performed echocardiography, blood biochemical testing, and apoptosis‐related detection after injecting adeno‐associated virus type 9 overexpressing TRF2 into aged mice. We found that TRF2 improved myocardial I/Post protection in vivo. Knockdown of TRF2 in a cardiomyocyte cell line (HL‐1) increased inflammatory factor release and aggravated mitochondrial and DNA damage in senescent myocardiocytes following hypoxic postconditioning (H/Post). TRF2 also inhibited activation of the cGAS/STING pathway by increasing mitophagy during H/Post in aged myocardiocytes. Moreover, TRF2 interacted with casein kinase 2 alpha 2 polypeptide (CSNK2A2) to regulate the dephosphorylation of FUN14 domain‐containing protein 1 (FUNDC1), which contributed to the protective effect of hypoxic postconditioning.

## Graphical abstract

Myocardial expression of telomeric repeat‐binding factor 2 (TRF2) was reduced in aged male mice and further downregulated following ischemic postconditioning (I/Post). Mechanistically, TRF2 confers cardioprotection by interacting with casein kinase 2 alpha 2 polypeptide (CSNK2A2) to modulate FUN14 domain‐containing protein 1 (FUNDC1) dephosphorylation, which enhances mitophagy and blunts cGAS‐STING signaling activation.

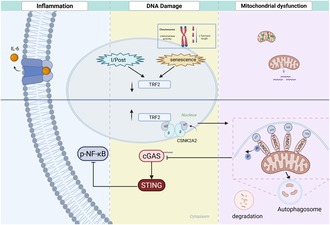

## 1 Introduction

The incidence of myocardial infarction has been progressively increasing over the years, and advanced age is an independent adverse prognostic factor in ischemic heart disease (Campos et al. 2016). Prompt restoration of coronary blood flow, such as through percutaneous coronary intervention, can enhance cardiac muscle perfusion. However, myocardial reperfusion may exacerbate heart damage, known as ischemia/reperfusion (I/R) injury. Furthermore, older patients show a larger myocardial infarct size, reduced myocardial salvage capacity, and increased extent of myocardial edema compared with younger patients (Park et al. 2021). Ischemic postconditioning (I/Post) refers to a substantial reduction in ischemic tissue damage achieved by repeated brief reperfusion episodes following prolonged ischemia but prior to full reperfusion. This phenomenon is generally attributed to the activation of endogenous protective pathways (Tsang et al. 2004). However, I/Post may lose its protective effect in aging hearts (Abete et al. 1997; Perez et al. 2016). Therefore, the underlying reasons for the diminished protective effect observed with I/Post need to be investigated, and novel therapeutic strategies aimed at mitigating myocardial I/R injury in older patients need to determined.

A telomere is a type of DNA protein complex composed of TTAGGG DNA tandem repeats and shelterin that covers the end of eukaryotic chromosomes and is recognized as the biological clock of cellular aging. Telomere shortening is a key biomarker of the onset of senescence (Feng et al. 1995; Christensen et al. 2009). Clinical studies have shown that a shorter telomere length in white blood cells is associated with a higher risk of ischemic heart disease (Scheller Madrid et al. 2016; Weischer et al. 2012). In addition to the telomere length, telomerase activity is also associated with aging and myocardial injury (Ait‐Aissa et al. 2019). Telomerase is a ribonucleic protease composed of RNA and protein, and its composition includes telomerase template RNA and telomerase reverse transcriptase (TERT). Additionally, telomerase‐related catalytic proteins are enzymes that can add telomere repeat sequences to the 3′ end of telomeres (Shay 2016). The overexpression of TERT can prolong the lifespan of mice and reduce the incidence of certain age‐related degenerative diseases (Beyer et al. 2016). After establishment of the I/R model, the infarct area of TERT in the mitochondria mice is larger than that of wild‐type mice (Ait‐Aissa et al. 2019). Six telomere‐binding proteins form the shelterin complex, which is responsible for the maintenance of telomere length and the suppression of the DNA damage response, and dysfunction of telomere shelterin drives telomere damage and cardiac dysfunction in ischemic heart disease (Zhang et al. 2022). One of the shelterin complex, repressor activator protein 1 (Rap1), regulated cardiac aging in mice and myocardial ischemia/reperfusion injury (Cai et al. 2021; Cai et al. 2020). Telomeric repeat binding factor 2 (TRF2) is a component of shelterin, which specifically binds to telomeric DNA. TRF2 is directly bound to telomere double‐stranded DNA, and loss or mutation can lead to DNA damage and telomere shortening, resulting in replicative aging (Mao et al. 2007). In primary cardiomyocytes, the knockdown of TRF2 leads to telomere shortening and cellular senescence, while overexpression of TRF2 mitigates oxidative stress damage induced by hydrogen peroxide (H_2_O_2_) in cardiomyocytes and preserves telomere length (Oh et al. 2003). In mice subjected to voluntary wheel running for 3 weeks, there was an increase in telomerase activity, elevated expression of TRF2, and a reduction in the expression of vascular apoptosis regulators (Werner et al. 2009). However, the potential role of TRF2 in ischemia/reperfusion injury in aged cardiomyocytes has not been elucidated.

Aging, mitochondrial dysfunction, and inflammation form a vicious cycle in I/R injury (Xu et al. 2025). In recent years, an increasing number of studies have shown that the cGAS‐STING pathway is an important driver in the relationship between these factors. Trap1‐mediated metabolic reprogramming increases lactate‐dependent H4K12la through HDAC3, promoting senescence‐associated secretory phenotype expression, vascular smooth muscle cell aging, and atherosclerosis (Li et al. 2024). A deficiency of glycerol‐3‐phosphate acyltransferase 4 provokes an endocardial endoplasmic reticulum stress response and enhances endoplasmic reticulum–mitochondrial communications. This process leads to mitochondrial DNA escape, which activates the cGAS‐STING pathway and affects heart development (Zhao et al. 2025).

However, few studies have reported the role of TRF2 in the relationships between aging, myocardial I/Post injury, and the cGAS‐STING pathway. Therefore, this study aimed to determine whether TRF2 provides protection against myocardial I/Post injury in the aged heart and to investigate whether the cGAS‐STING pathway is involved in this protection.

## 2 Methods

### 2.1 Animal Model, Experimental Design, and Echocardiography

The study protocol was approved by the Tianjin Medical University Laboratory Animal Ethics Committee. Male C57BL/6 mice (4 months) were provided by Vital River Laboratory Animal Technology Co. Ltd. (Beijing, China). The male mice were kept in a single cage until 18 months of age, reared under standard conditions, and had free access to clean drinking water and feed throughout the day. After intraperitoneal administration of sodium pentobarbital (40 mg/kg) anesthesia, the mice were placed in the supine position on the operating table. The chest was depilated and disinfected, and an incision was made between the third and fourth ribs on the left side of the sternum to expose the heart. The left anterior descending artery was identified between the left atrial appendage and pulmonary artery cone, and a suture needle with thread was passed through it at a distance of 2 mm from its origin. A pad was placed on the myocardium, and the left anterior descending artery was ligated with a single knot. After a 30‐min observation period, reperfusion occurred by loosening the thread for 10 s, followed by ligation for 10 s, repeated three times (I/Post group). In the sham group, the mice received the same surgical procedures as those in the I/Post group without ligation of the slipknot around the left anterior descending artery for 30 min. The chest was then closed and the wound was sutured. Reperfusion continued for an additional duration of 12 h, followed by various tests (see below).

In another experiment, mice were intravenously injected with 100 μL of adeno‐associated virus type 9 at a dose of 5 × 10^11^ vg, followed by a 3‐week feeding period. Subsequently, 28 days later, I/Post was conducted and the experimental method was performed as mentioned above.

A Mindray high‐frequency color Doppler ultrasound system (Shenzhen, China) with a 30‐MHz high frequency ultrasound probe was used to obtain ultrasound images of the left ventricle from the parasternal long‐axis view. The left ventricular ejection fraction (LVEF%) and fractional shortening (FS%) were calculated by the diastolic left ventricular diameter and systolic left ventricular diameter.

### 2.2 Cell Culture and Treatment

HL‐1 cells (i.e., mouse myoblasts) were purchased from the Shanghai Institute of Biochemistry and Cell Biology (Shanghai, China). The cells were cultured in DMEM supplemented with 10% fetal bovine serum and penicillin–streptomycin liquid at 37°C and 5% CO_2_.

To stimulate hypoxia/reoxygenation (H/R) injury in the cells, the medium was changed to Hanks' balanced salt solution (Gibco, USA), and the cells were cultured at 37°C in a hypoxic incubator (Billups‐Rothenberg, USA) filled with pre‐mixed gas (5% CO_2_ and 95% N_2_). Hypoxic postconditioning (H/Post) was performed after hypoxia was induced by exposing cells to 30 min of hypoxia and 30 min of reoxygenation for three cycles. After hypoxia or H/Post, the cells were transferred to normal culture medium and kept in an incubator for 6 h of reoxygenation.

Based on our team's previous research experience, H_2_O_2_ was used to accelerate the aging of the HL‐1 cell line. Various concentrations of H_2_O_2_ were added to DMEM for 4 h to induce the senescence of HL‐1 cells.

### 2.3 Cell Transfection

TRF2 shRNAs were purchased from Tsingke (Beijing, China). Two TRF2 shRNAs were mixed for transfection into cells. The sense sequences of TRF2 used in this study were as follows: GCUGCUUCAAGUACAAUGA and GCUGGGUGCUCAAGUUCUA. Full‐length sequences of TRF2, Flag‐TRF2, CSNAK2A2, Myc‐CSNA2A2, and FUNDC1ser17 were cloned into pcDNA3.1 to generate an overexpression vector. FUNDC1ser17A was mutated at the ser17 site to mimic dephosphorylation with the following primers: FUNDC1ser17A‐F, 5′‐TATGAAAGTGATGACGAC**GCT**TATGAAGTGTTG‐3′ and FUNDC1ser17A‐R, 5′‐ATCCAACACTTCATAAGCGTCGTCATCACTTTC‐3′. Lipofectamine 2000 reagent (Invitrogen) was used for cell transfection according to the manufacturer's instructions.

### 2.4 Senescence‐Associated β‐Galactosidase Assay

Senescence‐associated β‐galactosidase (SA‐β‐gal) staining was performed using an SA‐β‐gal staining kit (Beyotime, Shanghai, China) according to the manufacturer's instructions. Briefly, the cells were washed in phosphate‐buffered saline, fixed for 15 min at room temperature with fixative solution, and incubated overnight at 37°C with the staining solution mix. The percentage of senescent (SA‐β‐gal‐positive) cells was calculated in five randomly chosen fields of view for each sample.

### 2.5 Apoptosis Assay

Cell apoptosis was observed by flow cytometry following propidium iodide and fluorescein isothiocyanate‐conjugated Annexin V staining (Beyotime, China). Briefly, cells were suspended in binding buffer and stained in propidium iodide and fluorescein isothiocyanate‐Annexin V according to the manufacturer's instructions. The samples were analyzed by flow cytometry (Beckman, USA).

The terminal transferase dUTP nick‐end labeling (TUNEL) assay (Beyotime) was used to assess apoptosis of tissue. In brief, the sections were deparaffinized and hydrated by xylene and ethanol, followed by digestion with Proteinase K at 55°C for 1 h. The sections were then washed twice with ice‐cold phosphate‐buffered saline and incubated with TUNEL reaction mixture at 37°C for 1 h. Subsequently, the sections were stained with DAPI solution at room temperature for 5 min in the dark. The immunofluorescence images were obtained under an inverted fluorescence microscope (Leica, Wetzlar, Germany).

### 2.6 Mitochondrial and Lysosomal Staining

Mito‐Tracker Red (Beyotime) was diluted with anhydrous DMSO (Solarbio, Beijing, China), and the final concentration after dilution was 1 mM. A volume of 1 μL of this solution was added to 5 mL of cell culture medium. The concentration of the working solution was diluted to 200 nM.

Lyso‐Tracker Green (1 mM) was incubated in a 25°C water bath until it was completely dissolved for later use. A total of 1 μL of dissolved Lyso‐Tracker Red was added to 13.33 mL of cell culture medium and mixed. The final concentration of Lyso‐Tracker Red after dilution was 75 nM.

After the cell stimulation intervention was completed, the culture medium was aspirated with a gun, discarded, and added to pre‐incubated Mito‐Tracker Red working solution or Mito‐Tracker Red and Lyso‐Tracker Green mix working solution at 37°C. Each cell sample was reacted with the cells at 37°C for 30 min. The fluorescence was detected by an inverted fluorescence microscope (Leica).

### 2.7 Mitochondrial Membrane Potential Measurement

A mitochondrial membrane potential assay kit with JC‐1 (Beyotime) was used to detect the mitochondrial membrane potential. Cells were transferred to JC‐1 staining working solution and equal amounts of DMEM medium, and then incubated at 37°C in 5% CO_2_ for 20 min. After the incubation, the cells were washed twice with JC‐1 buffer at room temperature and then transferred to DMEM medium. The fluorescence of the cells was analyzed under an inverted fluorescence microscope (Leica).

### 2.8 Immunofluorescence Staining

Cells were inoculated into culture plates and 4% paraformaldehyde was added to the cells at room temperature for 15 min to maintain the cell morphology and integrity of the antigen. We then added 0.1% Triton X‐100 and allowed the cells to sit for 20 min followed by 10% BSA for 30 min to minimize nonspecific binding. The cells were incubated overnight with the corresponding primary antibody at 4°C, washed with phosphate‐buffered saline three times, and light was avoided to join two anti‐fluorescent secondary antibodies, with incubation for 60 min. The nuclear dye DAPI was added and allowed to sit for 5 min, resistance to quenching fluorescence microscopy after sealing seal tablets. The fluorescence of the cells was analyzed using an inverted fluorescence microscope (Leica).

### 2.9 Biochemical Indices Assays

Creatine kinase‐MB (CK‐MB) concentrations in the blood were also evaluated by an ELISA assay using a commercial kit (Solarbio) according to the manufacturer's instructions. Interleukin (IL)‐6 concentrations in blood homogenate were determined using the Mouse IL‐6 ELISA Kit (Beyotime) according to the manufacturer's instructions. Subsequently, the absorbance was measured using a microplate reader (Thermo Fisher Scientific, Massachusetts, USA).

### 2.10 Cell Viability Assay

Cells were seeded in 96‐well plates overnight and treated with various compounds. The CCK8 assay (Life Technologies Corporation, USA) was carried out after incubation of the cells for 3 days. The absorbance (optical density) was read at a wavelength of 450 nm for CCK8 on an ELISA plate reader (Thermo Fisher Scientific).

### 2.11 Immunoblotting

Samples were lysed in lysis buffer with phenylmethylsulfonyl fluoride buffer for 30 min on ice and were quantified using a BCA Protein Assay Kit (Beyotime). After separation using sodium dodecyl sulfate–polyacrylamide gel electrophoresis, proteins in the gels were transferred onto polyvinylidene difluoride membranes. The membranes were then blocked with non‐fat milk for 1 h at room temperature and incubated with primary antibody at 4°C overnight, followed by washing and incubation with secondary antibody conjugated with horseradish peroxidase for 1.5 h at room temperature. Signal were detected with the Amersham Imager 600 (Cytiva, Sweden) and quantified by ImageJ Software.

The following primary antibodies were used in this study: TRF2, Bax, LC3B (Sigma, MO, USA), FUNDC1 (Affinity, Jiangsu, China), Bcl‐2, GAPDH, Flag, c‐Myc, P62, p‐NF‐κB, IL‐6, SIRT1, PCG1α (Proteintech, China), and P53 (Cell Signaling Technology, MA, USA). Additionally, FUNDC1ser17A polyclonal antibody was generated by immunizing rabbits from our lab. The secondary antibodies used for western blotting were as follows: HRP goat anti‐mouse immunoglobulin (Ig) G, HRP goat anti‐rabbit IgG, Alexa fluor 488‐labeled Donkey anti‐rabbit IgG, and Alexa fluor 594‐labeled Donkey anti‐mouse IgG antibody (Proteintech).

### 2.12 Immunoprecipitation

Immunoprecipitation was conducted by using an immunoprecipitation kit (Beyotime) according to the manufacturer's instructions. Briefly, after being treated as described in the text, 500 μg of total cellular proteins were extracted and used for immunoprecipitation by adding protein A/G magnetic beads bound to TRF2 antibody or Flag antibody at 4°C overnight. The immunoprecipitated proteins were analyzed by western blot analysis with anti‐FUNDC1 or c‐Myc antibody.

### 2.13 Subcellular Fractionation

Nuclear proteins were extracted using the Nuclear and Cytoplasmic Protein Extraction Kit (Beyotime, Shanghai, China) according to the manufacturer's instructions. Mitochondria were extracted using the Cell Mitochondria Isolation Kit (Beyotime) by following the manufacturer's guidelines.

### 2.14 Telomere Length Assay

Genomic DNA was isolated and purified from the samples using the TIANamp Genomic DNA Kit (Tiangen, Beijing, China). The telomere length was determined using a validated quantitative polymerase chain reaction (qRT‐PCR)‐based analysis compared with the reference gene 36B4 on the CFX96 Real‐time System instrument (Bio‐Rad, CA, USA). In brief, the method included amplifying the telomeres of the experimental DNA extracted from venous blood against a single copy gene and comparing the ratio of the telomere to single copy gene concentration with that of a reference DNA of known telomere length. The primer sequences were as follows: Tel‐F, 5′‐CGGTTTGTTTGGGTTTGGGTTTGGGTTTGGGTTTGGGTT‐3′ and Tel‐R, 5′‐GGCTTGCCTTACCCTTACCCTTACCCTTACCCTTACCCT‐3′; and 36B4‐F, 5′‐ACTGGTCTAGGA CCCGAGAAG‐3′ and 36B4‐R, 5′‐TCAATGGTGCCTCTGGAGATT‐3′. The PCR for telomeres involved one cycle of 95°C for 3 min and 20 cycles of 95°C for 15 s. The 36B4 cycling profile was followed by one cycle of 95°C for 3 min and 30 cycles of 95°C for 15 s.

### 2.15 Quantitative Real‐Time PCR

Total RNA was extracted with Trizol (Solarbio) and chloroform, precipitated with isopropyl alcohol, washed with 75% ethanol, dissolved in diethyl pyrocarbonate‐treated water, dried, and dissolved in RNase‐free water. RNA was reverse transcribed with a reverse transcriptase kit (Takara, Kusatsu, Japan) and subjected to qRT‐PCR using SYBR Green (Takara) according to the manufacturer's instructions. The primers of TERT were as follows: mTERT F, 5′‐ATGGCGTTCCTGAGTATG‐3′ and mTERT R, 5′‐TTCAACCGCAAGACCGACAG‐3′. The qRT‐PCR was carried out on the CFX96 Real‐time System instrument (Bio‐Rad). GAPDH was used as an endogenous control.

Each sample was run in triplicate. Comparative quantification was performed using the 2^−ΔΔCt^ method.

### 2.16 Statistical Analysis

Measurement data are expressed as the mean ± standard deviation. Differences between two groups with normally distributed data were evaluated using Student's *t*‐test. More than two groups were compared using one‐way analysis of variance followed by Tukey's multiple comparisons test. The cell growth viability was performed using two‐way repeated‐measures ANOVA followed by Bonferroni's multiple comparison test. All the analyses were performed using GraphPad Prism version 8.0 software (GraphPad Software, California, USA). *p* < 0.05 indicates statistical significance.

## 3 Results

### 3.1 Aggravation of I/Post Injury in Aged Mice Is Related to Decreased TRF2 Expression

**FIGURE 1:**
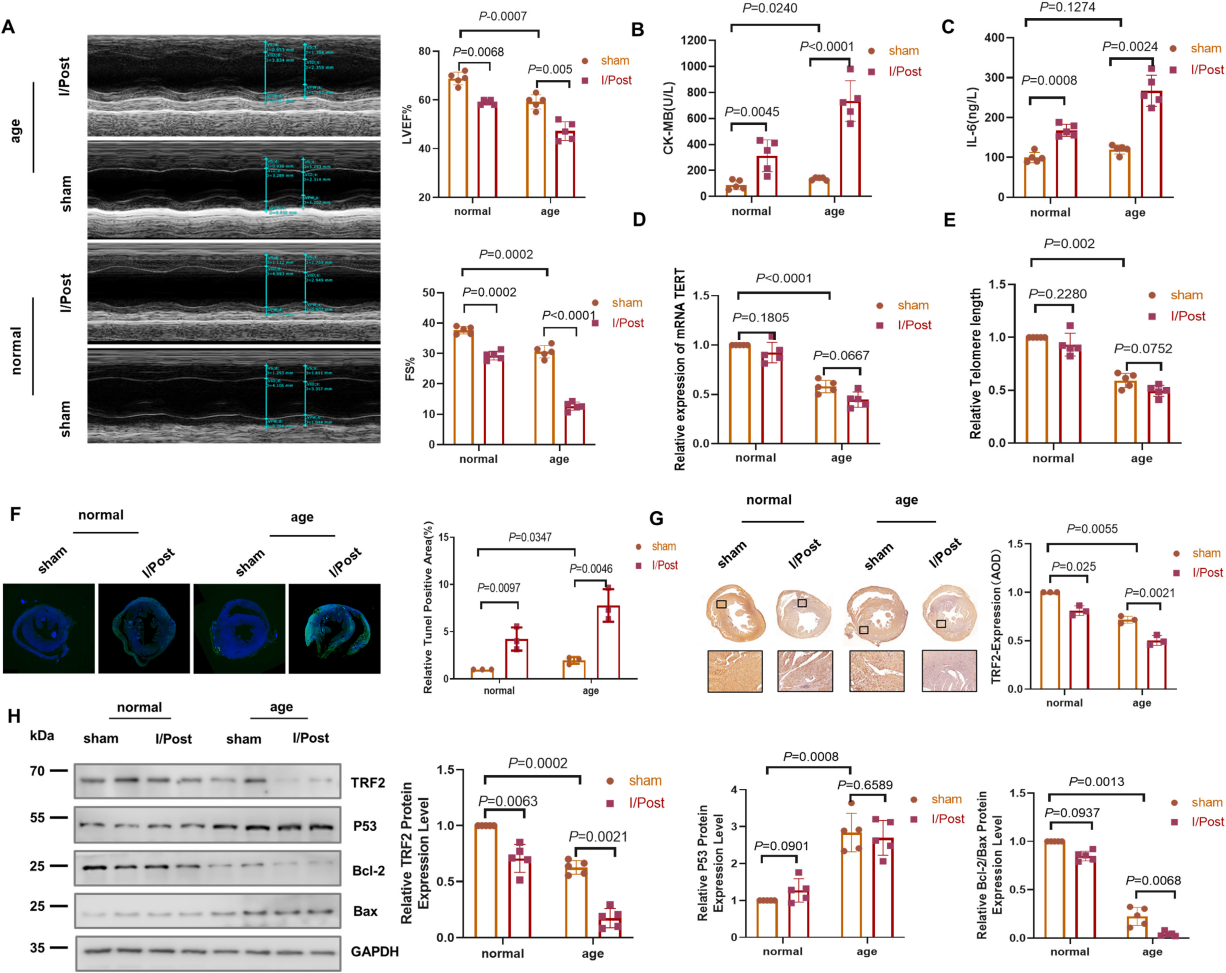
Failure to improve injury during ischemic postconditioning in elderly mice is associated with decreased expression of TRF2. (A) Echocardiography shows LVEF% and FS% 12 h after the operation (*n* = 5). (B) CK‐MB expression detected by ELISA of venous blood of mice (*n* = 5). (C) IL‐6 concentrations detected by ELISA of venous blood in mice (*n* = 5). (D) TERT mRNA expression by qRT‐PCR (*n* = 5). (E) Telomere length detected by RT‐PCR (*n* = 5). (F) TUNEL staining of myocardial apoptosis in each treatment group (*n* = 3). (G) Immunohistochemistry of TRF2 protein expression in myocardial tissue (*n* = 3). (H) Western blot analysis of TRF2, Bcl2, Bax, and P53 protein expression in the myocardium of adult aged mice in the sham and I/Post groups (*n* = 5).

The mouse models of I/Post in adult (4 months) and aged mice (18 months) were established. The LVEF% and FS% of mice in each treatment group 12 h after the operation were detected by echocardiography. The LVEF% and FS% in the I/Post group were significantly lower in adult mice, and this reduction was more obvious in aged mice than in mice in the sham group (Figure 1A). CK‐MB and IL‐6 concentrations were increased in aged mice following I/Post (Figure 1B,C). The mRNA levels encoding TERT are basically consistent with overall telomerase activity. Therefore, TERT mRNA levels can be used to measure telomerase activity (Oshita et al. 2000). In this study, TERT mRNA expression levels in the myocardium were lower in aged mice in the I/Post group than in those in the sham group. TERT mRNA expression was also decreased but this was not significant (Figure 1D). Similarly, the telomere length in venous blood of aged mice in the I/Post group was decreased, but there was no significant difference between the sham and I/Post groups (Figure 1E). TUNEL staining showed that the degree of myocardial apoptosis in aged mice was greater than that in adult mice in the I/Post group (Figure 1F). Immunohistochemistry also showed that TRF2 protein expression was decreased in aged mice in the I/Post group (Figure 1G). P53 protein expression levels were higher in aged mice and TRF2 protein expression levels were lower in aged mice than in adult mice. TRF2 protein expression levels in aged mice were also lower in the I/Post group than in sham group. The ratio of Bcl2/bax was slightly lower in adult mice in the I/Post group than in those in the sham group. Additionally, the ratio of Bcl2/bax in aged mice was lower in the I/Post group than in the sham group (Figure 1H).

In summary, the myocardial contractile function of aged mice was decreased after I/Post, the expression of myocardial enzymes and inflammatory factors was increased, the infarct area of myocardial tissue was increased, telomerase activity and telomere length were decreased, and TRF2 expression was decreased in aged mice and further decreased after I/Post. Therefore, the injury of aged mice after I/Post may be related to decreased TRF2 expression.

### 3.2 TRF2 Overexpression Reduces I/Post Injury in Aged Mice

**FIGURE 2:**
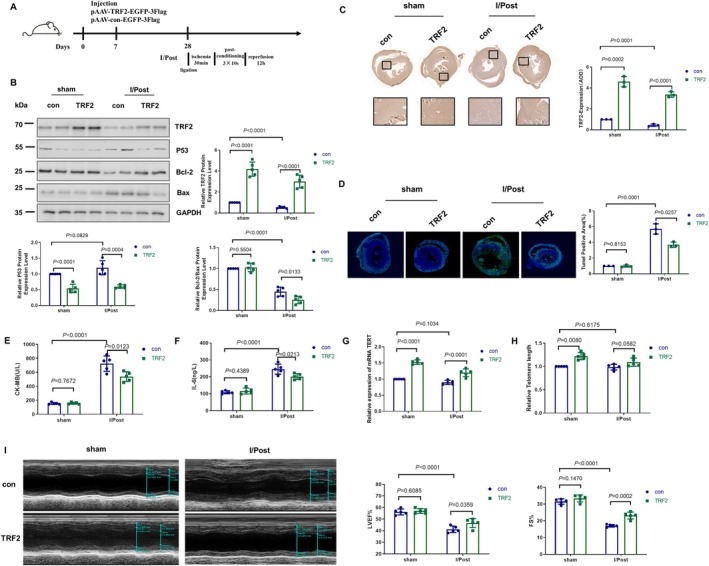
TRF2 reduces I/Post injury in aged mice. (A) Schema of mouse experiments using the AAV model. (B) Western blot analysis of TRF2, Bcl‐2, bax, and P53 protein expression in the myocardium of aged mice after I/Post (*n* = 5). (C) Immunohistochemistry of TRF2 protein expression in the myocardium in each treatment group (*n* = 3). (D) TUNEL staining of myocardial apoptosis in each treatment group (*n* = 3). (E) CK‐MB concentrations by ELASA (*n* = 5). (F) IL‐6 concentrations by ELISA. (G) TERT mRNA expression by qRT‐PCR (*n* = 5). (H) Telomere length by qRT‐PCR (*n* = 5). (I) Echocardiography shows the LVEF% and FS% (*n* = 5).

After 1 week of adaptive feeding, 18‐month‐old mice were injected with adeno‐associated virus (AAV) in the tail vein. The AAV model was established after 3 weeks of continuous feeding (Figure 2A). TRF2 protein expression was significantly increased after transfection with AAV as shown by western blotting and immunohistochemistry (Figure 2B,C). We found that P53 protein expression was decreased after overexpression of TRF2, and the ratio of Bcl‐2/Bax in the overexpressed TRF2 group was decreased after I/Post. TUNEL staining showed that the proportion of myocardial apoptosis decreased after overexpression of TRF2 in the aged heart (Figure 2D). CK‐MB and IL‐6 concentrations in the AAV‐TRF2 group were lower than those in the control AAV group (Figure 2E,F). TERT mRNA expression in the AAV‐TRF2 group was lower than that the control group, but telomere length was not different between the groups (Figure 2G,H). The LVEF% and FS% were measured by ultrasonography at 12 h after reperfusion. When overexpression of TRF2 increased the values of LVEF% and FS% that improved the I/Post injury of aging heart (Figure 2I).

### 3.3 Failure to Ameliorate H/R Injury by H/Post Treatment in Senescent Cardiomyocytes Is Associated With Decreased TRF2 Expression

**FIGURE 3:**
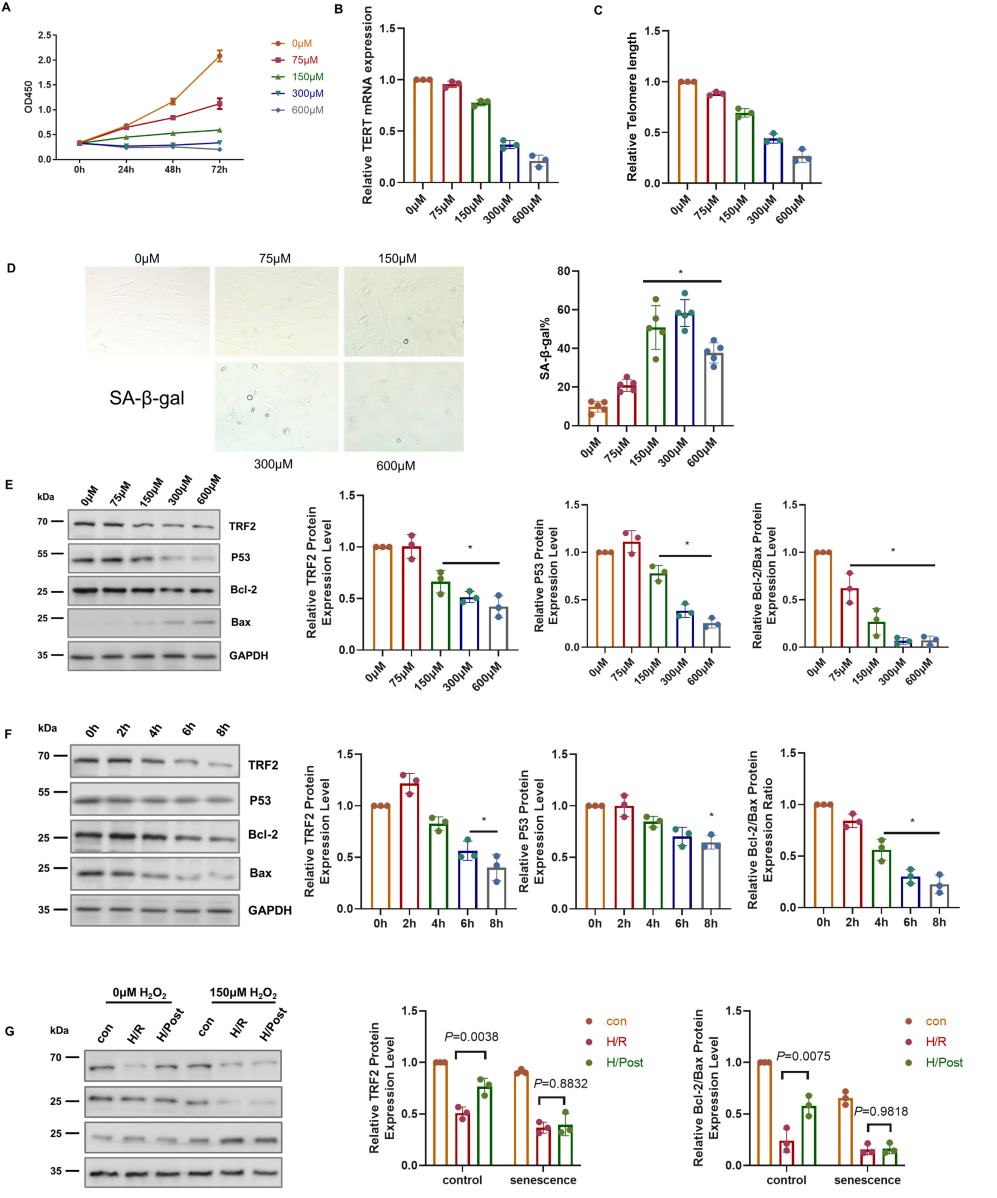
H/Post of aging cardiomyocytes does not improve H/R injury, which is related to decreased TRF2 protein expression. (A) HL‐1 cells were treated with 0, 75, 150, 300, and 600 μM H_2_O_2_ for 4 h, and the CCK‐8 assay was used to detect myocardial cell activity after 24, 48, and 72 h (*F*_time_ = 705.3, *p* < 0.0001; *F*_concentration_ = 417.9, *p* < 0.0001). (B) TERT mRNA expression levels of HL‐1 cells treated with 0, 75, 150, 300, and 600 μM for 4 h, after 24 h measured by qRT‐PCR (*F* = 4550.4, *p* < 0.0001). (C) Telomere length by qRT‐PCR (*F* = 1143.7, *p* = 0.0047). (D) SA‐β‐gal staining of HL‐1 cells treated with 0, 75, 150, 300, and 600 μM after 24 h (*p < 0.05 vs. 0 μM group). (E) HL‐1 cells were treated with different concentrations of H_2_O_2_ for 4 h, and after 24 h, protein was collected. Western blot analysis of P53, TRF2, Bcl2, and Bax relative protein expression levels is shown (*p < 0.05 vs. 0 μM group). (F) Western blot analysis of P53, TRF2, Bcl2, and Bax relative protein expression levels in HL‐1 cells treated after different periods of hypoxia (*p < 0.05 vs. 0 h group). (G) Western blot analysis of P53, TRF2, Bcl2, and Bax protein expression after H/R and H/Post in HL‐1 cells treated with 0 μ or 150 μM H_2_O_2_.

HL‐1 cells were seeded into 96‐well plates at a density of 5 × 10^4/L. The cells were then replaced with media containing different concentrations of H_2_O_2_, with the final concentrations of 0, 75, 150, 300, and 600 μM for 4 h and then changed to normal culture medium. Cell growth was detected by the CCK‐8 assay after 24, 48, and 72 h. Optical density values at 450 nm were recorded with three multiple holes in each group. We found that the viability of cardiomyocytes was not increased when treated with H_2_O_2_ concentrations > 150 μM with an increase in the intervention time and concentration (Figure 3A). After being treated with different concentrations of H_2_O_2_ for 4 h, RNA and DNA were extracted after 24 h. By detecting TERT mRNA level and quantifying telomere length, we demonstrated that different concentrations of H_2_O_2_ exert dose‐dependent deteriorative effects on senescence progression and telomere impairment in HL‐1 cells (Figure 3B,C). Sequentially, senescent cell staining with SA‐β‐gal after treatment with different H_2_O_2_ concentrations for 24 h showed that the proportion of cells dyed blue increased with an increase in treatment concentration. Bur, when treated at a concentration of 600 μM, most cells underwent apoptosis, while the number of senescence‐positive cells decreased instead (Figure 3D). Twenty‐four hours after H_2_O_2_ treatment, when the final concentration was > 150 μM, TRF2 protein expression decreased, P53 protein expression increased, and Bcl2/Bax protein expression decreased as H_2_O_2_ concentrations increased (Figure 3E). Additionally, we found that Bcl2/Bax protein expression decreased with an increase in the hypoxia time of the cells. Furthermore, TRF2 protein expression was increased following 2 h of hypoxia but was obviously reduced following exposure to hypoxia for more than 6 h. Protein expression of P53 was not remarkably decreased with an increase in the hypoxia time (Figure 3F). When H/R and H/Post were performed 24 h after treatment with 150 μM H_2_O_2_, we found that Bcl2/Bax protein expression in control HL‐1 cells was higher after H/Post than that after H/R, but there was no significant difference between the two groups of cardiomyocytes. Additionally, TRF2 protein expression was higher after H/Post in normal cardiomyocytes than that after H/R, with no significant difference in aged cardiomyocytes. These findings indicated that the failure of H/Post to improve H/R injury in aged cardiomyocytes may be related to decreased TRF2 protein expression (Figure 3G).

### 3.4 Knockdown of TRF2 Increases DNA Damage and Causes Release of Inflammatory Factors and Mitochondrial Damage During H/Post Treatment of Aged Cardiomyocytes

**FIGURE 4:**
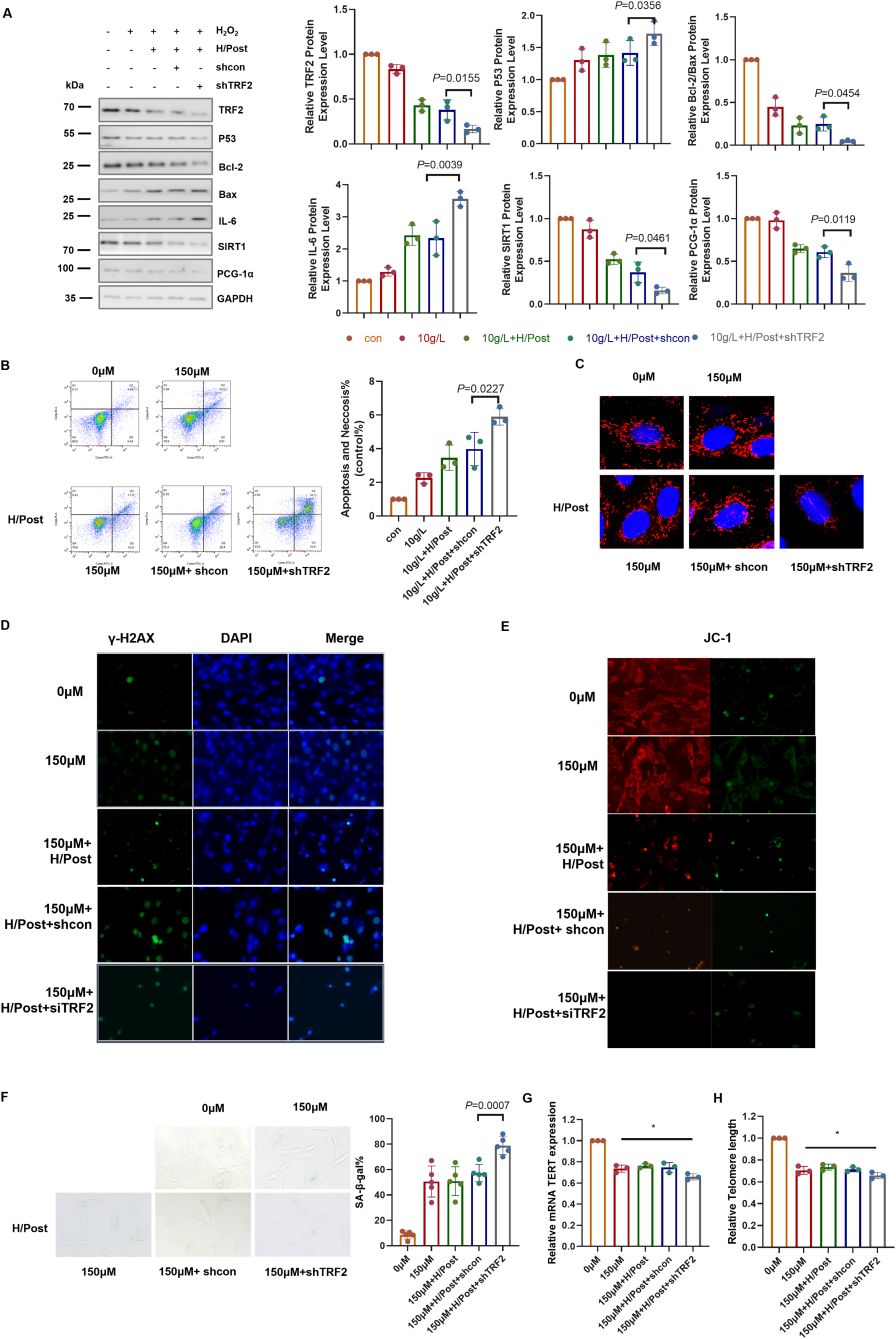
Knockdown of TRF2 increases H/Post injury of aged cardiomyocytes. The HL‐1 cell line was treated with 150 μM H_2_O_2_ for 4 h. After 24 h, the cells were transfected with control vector or shTRF2, followed by the H/Post experiment after another 24 h. (A) Western blot analysis of TRF2, P53, Bcl‐2/Bax, IL‐6, SIRT1, and PCG‐1α protein expression in each treatment group. (B) Flow cytometry of cellular apoptosis and the necrosis ratio. (C) Mitochondrial tracing staining to indicate mitochondrial morphology. (D) Immunofluorescence of γ‐H2AX staining in each treatment group. (E) Fluorescence of the JC‐1 probe as observed by fluorescence microscopy. (F) The degree of cellular senescence shown by SA‐β‐gal dye. (G) TERT mRNA expression levels by qRT‐PCR（*p < 0.05）. (H) Telomere length by qRT‐PCR (*p < 0.05).

To achieve a higher transfection efficiency and increase the stability of the models of hypoxia and accelerated aging, we used the HL‐1 cell line to investigate the function and mechanism of TRF2. HL‐1 cells were treated with 150 μM H_2_O_2_ for 4 h and transfected with control vector or shTRF2. The H/Post assay was performed 24 h later, and protein was collected for a western blot analysis. The increased expression of the apoptosis‐related proteins Bcl‐2/Bax suggested that H/Post damage of aged cardiomyocytes increased after TRF2 knockdown. We also found that protein expression of the inflammatory factor IL‐6 was increased, and protein expression of the mitochondrial function‐related proteins SIRT1 and PCG‐1α were decreased (Figure 4A). Flow cytometry of the other part of HL‐1 cells also showed that the percentage of apoptosis of cardiomyocytes increased after TRF2 knockdown at H/Post (Figure 4B). Mitochondrial tracing staining showed that after TRF2 knockdown followed by H/Post, the mitochondrial damage was further exacerbated (Figure 4C). Cell immunofluorescence showed that after TRF2 knockdown, the nuclear γ‐H2AX staining ratio was increased and DNA damage was increased at H/Post (Figure 4D). Additionally, microscopy showed that the proportion of green fluorescence and mitochondrial damage was increased in cardiomyocytes at H/Post after TRF2 knockdown as shown by the mitochondrial JC‐1 probe (Figure 4E). Senescence staining showed that after knocking down TRF2 and performing H/Post, the proportion of senescent cells was increased (Figure 4F). Regarding telomerase and telomere length, the shortening effect of accelerated aging by H_2_O_2_ was significant (Figure 4G,H). These findings suggested that knockdown of TRF2 increased DNA damage, and the release of inflammatory factors and mitochondrial damage were more pronounced after H/Post treatment of aged cardiomyocytes.

### 3.5 TRF2 Inhibits cGAS/STING Pathway Activation by Promoting Mitochondrial Autophagy Following H/Post

**FIGURE 5:**
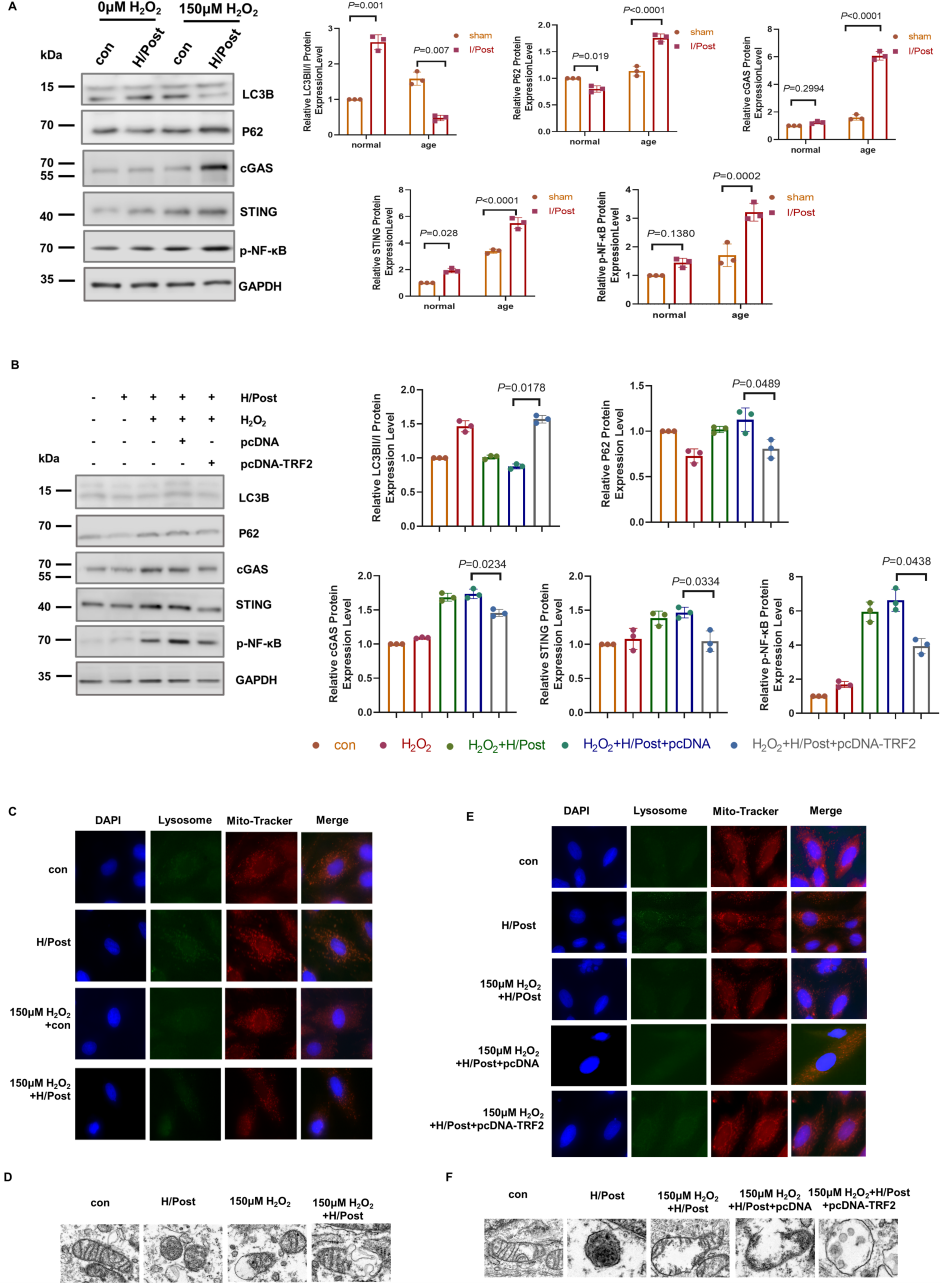
TRF2 inhibits cGAS/STING pathway activation by promoting mitochondrial autophagy following H/Post. (A) HL‐1 cells were treated with 150 μM H_2_O_2_ for 4 h, and H/Post was performed after 24 h. Western blot analysis of LC3BII/I, P62, cGAS, STING, and p‐NF‐κB protein expression is shown. (B) Hl‐1 cells were treated with 150 μM H_2_O_2_ and then transfected with pcDNA or pcDNA‐TRF2. Twenty‐four or 48 h after H/Post, protein expression was analyzed by western blotting. (C–F) A mitochondrial lysosomal immunofluorescence co‐staining experiment and electron microscopic showed the occurrence of mitophagy.

The above‐mentioned results indicated that TRF2 expression was decreased in aged mice after I/Post and in aged cardiomyocytes after H/Post. Moreover, after TRF2 was knocked down in aged cardiomyocytes, the levels of DNA damage, mitochondrial damage, and inflammation in cardiomyocytes were increased. Mitophagy reduces the release of mitochondrial DNA (mtDNA) by eliminating damaged mitochondria, the inhibition of mitophagy may activate the cGAS/STING pathway (Jiménez‐Loygorri et al. 2024). Therefore, we further examined the role of TRF2 in mitophagy and the cGAS/STING pathway. HL‐1 cells were treated with 150 μM H_2_O_2_ for 4 h and then switched to normal medium for 24 h under H/Post. In normal cardiomyocytes, H/Post increased the LC3BII/I (LC3B‐I appeared as an upper band, whereas LC3B‐II was detected as a lower band) ratio and decreased P62 expression, which suggested that mitophagy was increased, whereas in aged cardiomyocytes, H/Post impaired mitophagy. We also found that the cGAS/STING pathway was further activated in aged cardiomyocytes after H/Post. Additionally, p‐NF‐κB protein expression was increased in aged cardiomyocytes after H/Post (Figure 5A). HL‐1 cells were then treated with 150 μM H_2_O_2_ for 4 h and transfected with pcDNA and pcDNA‐TRF2 after 24 h. H/Post was performed another 24 h later. The level of mitophagy was increased and the cGAS/STING pathway and p‐NF‐κB protein expression were inhibited in aged cardiomyocytes after H/Post treatment (Figure 5B). Exogenous inhibition of mtDNA release by ethidium bromide (EtBr) reduces cGAS/STING activation following H/Post in aged cells, whereas TRF2 knockdown abrogates this protective effect (Figure S1). To directly assess mitophagy after H/Post, a mitochondrial lysosomal immunofluorescence co‐staining experiment was conducted. Immunofluorescence showed that in normal cardiomyocytes, mitophagy was increased, while autophagy was blocked in aged cardiomyocytes after H/Post (Figure 5C). Under electron microscopy, normal mitochondria exhibit a regular, elongated or oval shape with intact double membranes, after H/Post in normal cardiomyocytes mitochondria are surrounded by double‐membraned autophagosomal structures, forming mitophagosomes, while in aged cardiomyocytes following H/Post, mitochondrial membrane becomes discontinuous and ruptured. In aged cardiomyocytes following H/Post, damaged mitochondria displayed prominent vacuolation, disruption of the outer mitochondrial membrane, and irregular mitochondrial contours (Figure 5D). When overexpression TRF2, the level of mitophagy was increased (Figure 5E,F).

### 3.6 TRF2 Interacts With CSNK2A2 to Regulate the Phosphorylation Level of FUNDC1

**FIGURE 6:**
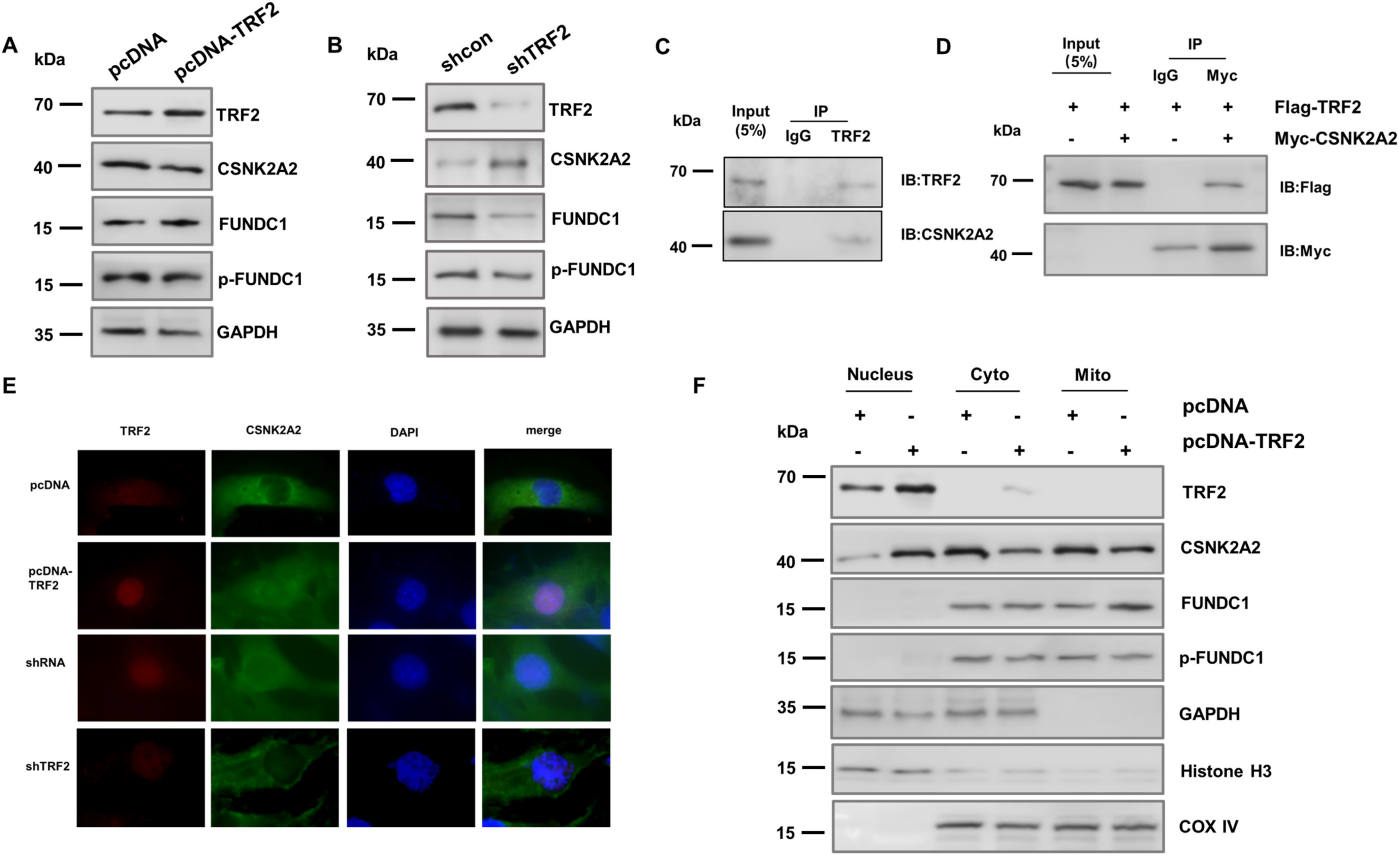
TRF2 interacts with CSNK2A2 to regulate the dephosphorylation of FUNDC1. (A) After transient pcDNA‐TRF2 or empty vector transfection in the HL‐1 cell line, western blotting was performed. CSNK2A2, FUNDC1, and p‐FUNDC1 protein expression is shown. (B) CSNK2A2, FUNDC1, and p‐FUNDC1 protein expression after transfection of shTRF2 or control vector into the HL‐1 cell line as shown by a western blot. (C) Using HL‐1 cells, endogenous immunoprecipitation showed an interaction between TRF2 and CSNK2A2. (D) HL‐1 cells were co‐transfected with Flag‐TRF2 and Myc‐CSNK2A2, and then cells were lysed and immunoprecipitated with anti‐Flag antibody 24 h after transfection. (E) Immunofluorescence images show that the changes in expression and positioning of CSNK2A2 protein after overexpressing TRF2. (F) CSNK2A2, FUNDC1, and p‐FUNDC1 protein expression in the nucleus, cytoplasm, and mitochondria as shown by a western blot.

The above‐mentioned results suggested that knocking down TRF2 in aging cardiomyocytes inhibited mitophagy and increased myocardial injury. To further examine the mechanism of these findings, we used the BioGRID website to predict TRF2‐interacting proteins and conducted interaction screening with mitochondrial autophagy‐related genes. We identified casein kinase 2 alpha 2 polypeptide (CSNK2A2) as a potential interact gene regulated by TRF2. CSNK2A2 is the primary polypeptide of tyrosine kinase 2 (CK2), which phosphorylates serine and threonine residues on proteins, including the mitophagy pathway‐related gene FUN14 domain‐containing protein 1 (FUNDC1) (Kim and Koh 2023). After using transiently infected pcDNA‐TRF2 in HL‐1 cells, we observed an increase in TRF2 protein expression, a decrease in CSNK2A2 protein expression, and an increase in FUNDC1 protein expression by western blotting. However, p‐FUNDC1 ser17 protein expression was decreased (Figure 6A). After we knocked down TRF2, CSNK2A2 protein expression was increased, FUNDC1 protein expression was decreased, and p‐FUNDC1 protein expression was also decreased (Figure 6B). We performed endogenous immunoprecipitation and found that TRF2 interacted with CSNK2A2 in HL‐1 cells (Figure 6C). Additionally, after transfecting Flag‐TRF2 and Myc‐CSNK2A2 plasmids into the HL‐1 cell line, we further validated the interaction between TRF2 and CSNK2A2, but not with the IgG control (Figure 6D). Using cellular immunofluorescence, we found that overexpressing TRF2 increased CSNK2A2 protein expression in the nucleus, while knocking down TRF2 reduced its expression (Figure 6E). By isolating nuclear and cytoplasmic proteins and extracting mitochondrial proteins, followed by western blotting, we found that TRF2 affected the distribution of CSNK2A2. However, TRF2 increased FUNDC1 protein expression but did not enhance its phosphorylation level (Figure 6F).

### 3.7 TRF2 Improves H/Post Injury of Aging Cardiomyocytes by Regulating CSNK2A2/FUNDC1

**FIGURE 7:**
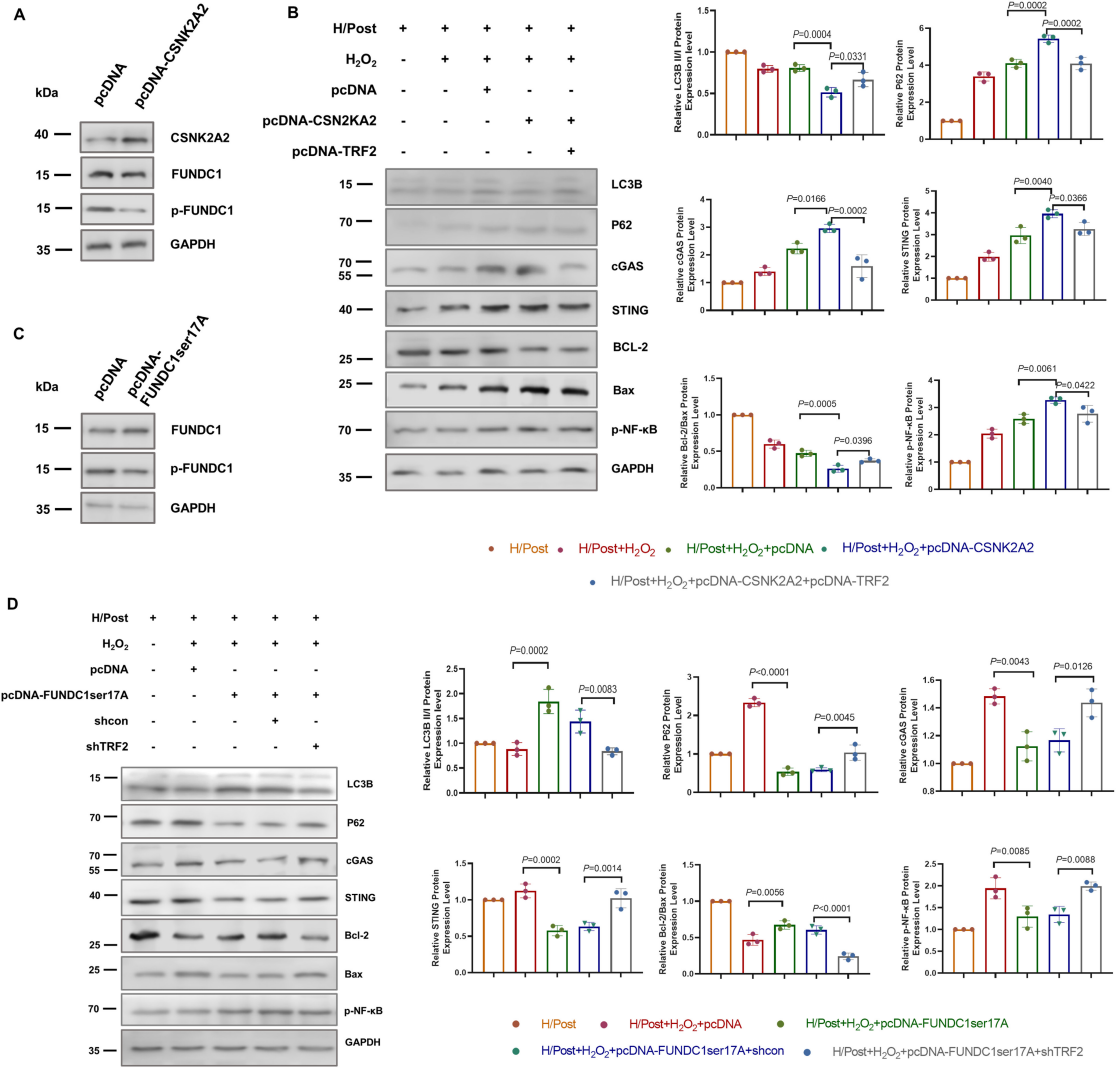
TRF2 improves H/Post injury of aging cardiomyocytes by regulating CSNK2A2/FUNDC1. (A) Western blot of CSNK2A2, FUNDC1, and p‐FUNDC1 protein expression with overexpression of CSNK2A2. (B) Western blot analysis of LC3B, P62, cGAS/STING, Bcl‐2/Bax, and p‐NF‐κB protein expression after transfection of CSNK2A2 and co‐transfection of CSNK2A2 and TRF2 in aged cardiomyocytes following H/Post. (C) Western blot of FUNDC1 and p‐FUNDC1 protein expression with overexpression of FUNDC1ser17A. (D) Western blot analysis of LC3B, P62, cGAS/STING, Bcl‐2/Bax, and p‐NF‐κB protein expression after transfection of FUNDC1ser17A and co‐transfection of FUNDC1ser17A and shTRF2 in aged cardiomyocytes following H/Post.

After overexpressing CSNK2A2 in cardiomyocytes, FUNDC1 and p‐FUNDC1 protein expression was increased (Figure 7A). After H/Post, when CSNK2A2 was overexpressed, the ratio of Bcl‐2/Bax protein expression decreased and the level of autophagy in cardiomyocytes was decreased. Furthermore, the cGAS/STING pathway and p‐NK‐κB protein expression were activated. However, co‐transfecting TRF2 improved mitophagy levels, inhibition of the cGAS/STING pathway, and myocardial injury caused by overexpressing CSNK2A2 (Figure 7B). A FUNDC1 overexpression plasmid was constructed, and serine at the 17th amino acid was mutated to alanine to simulate the dephosphorylation of FUNDC1. We detected FUNDC1 overexpression and the dephosphorylation effect of FUNDC1ser17 by cell transfection and western blotting (Figure 7C). When FUNDC1ser17A was overexpressed in aged cardiomyocytes and H/Post was performed, mitophagy was activated and the cGAS/STING pathway and p‐NK‐κB protein expression were inhibited; therefore H/Post injury was relieved. With knockdown of TRF2, the level of mitophagy decreased, the cGAS/STING pathway was activated, and H/Post injury was aggravated (Figure 7D).

## 4 Discussion

**FIGURE 8:**
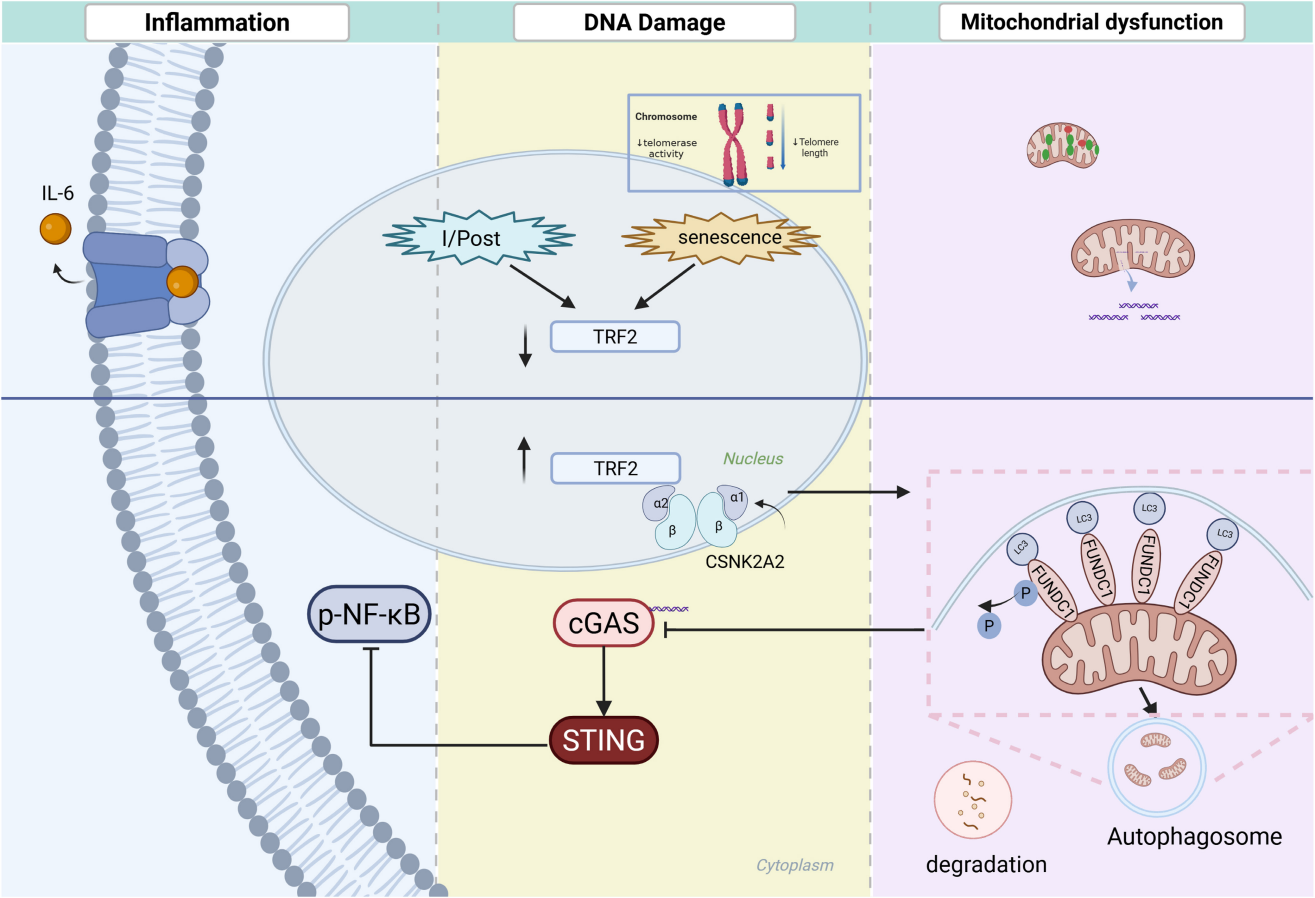
Schematic diagram showing the mechanism by which TRF2 alleviates myocardial I/Post injury in aged myocardiocytes.

The findings from our study suggest TRF2 protein expression was decreased in aged mice and further decreased after I/Post; TRF2 overexpression reduces I/Post injury in aged mice. The inability of aging myocardiocytes after I/Post to exert a protective effect against I/R injury was related to decreased TRF2 expression, inhibited mitophagy and activated cGAS/STING pathway. Overexpression of TRF2 activates the mitophagy pathway and this is related to its interaction with CSNK2A2, which reduces the phosphorylation of FUNDC1. This process inhibits the cGAS/STING pathway and inflammation, finally alleviating I/Post injury in aged myocardiocytes (Figure 8).

We previously showed that H/Post in aging cardiomyocytes did not improve H/R injury (Zhang et al. 2019). This previous study suggested the importance of identifying new research targets and pathways to improve I/R injury in older patients. Telomere attrition, telomerase deficiency, and cardiovascular disease increase with age (de Lange 2018). Telomerase deficiency predisposes the heart to I/R injury, and leukocyte telomere length is associated with the development of coronary heart disease (Chen et al. 2023). In the current study, we focused on TRF2, which is a telomere DNA–protein complex, and it protects the ends of chromosomes and directly binds to telomere double‐stranded DNA. In in vivo and in vitro *experiments*, we found that TRF2 expression was decreased in senescent cardiomyocytes after I/Post and H/Post. After overexpression of TRF2 by injection of AAV9 in vitro, the injury of I/Post in aged cardiomyocytes was alleviated. After knocking down TRF2 in myocardial cells, the injury of H/Post in aged myocardial cells was increased. TRF2 has been reported to prevent telomere shortening, restore cardiomyocyte size and sarcomere density, and improve cell survival in Duchenne muscular dystrophy cardiomyocytes (Eguchi et al. 2023). A mutant TRF2 protein (TRF2^T188A^) induced persistent telomere damage and senescence in vascular smooth muscle cells (Uryga et al. 2021). TRF2 is decreased in the brain of mice submitted to post‐hypoxic stress, and downregulation of TRF2 expression in hippocampal neural cells of unchallenged mice triggers an excitotoxicity‐like phenotype, including glutamate overexpression and behavioral alterations (Gao et al. 2024). In our study, TRF2 also played an important part in myocardial I/R injury, especially in injury of the aging heart.

Population aging is increasingly recognized as a major global challenge. Several studies have shown that mitochondrial damage and inflammation are the essential mechanisms of aggravated damage of I/R injury following aging. The activation of cGAS/STING signaling is also associated with this process. Therefore, we investigated the level of mitophagy, which is considered a beneficial metabolic event and contributes to the maintenance of intracellular homeostasis in most cells of cardiovascular origin (Bravo‐San Pedro et al. 2017). We found that in aged cardiomyocytes, the level of mitophagy was reduced and the cGAS‐STING pathway was activated following H/Post. TEF2 has been shown to promote the autophagic process in a tumor cell line (Iachettini et al. 2023). In our study, TRF2 inhibited cGAS/STING pathway activation by promoting mitochondrial autophagy and reducing mtDNA following H/Post.

In this study, proteins that might interact with TRF2 were screened out using BioGRID, and interactive screening was conducted with genes related to mitochondrial autophagy. We found that a coexisting gene was CSNK2A2, which is the primary polypeptide of CK2. CSNK2A2 encodes an enzyme that phosphorylates a large number of substrates and regulates numerous cellular processes, such as cell cycle progression, apoptosis, and transcription (Litchfield 2003). CSNK2A2 also interacts with multiple genes and miRNAs in a pathway controlling telomere length and CHD (Gao and Wang 2006). In our study, TRF2 interacted with CSNK2A2 in the nucleus, and CSNK2A2 expression in mitochondria was decreased. After overexpressing CSNK2A2 in aged cardiomyocytes and then undergoing H/Post, the level of mitophagy in cardiomyocytes was decreased and the cGAS/STING pathway was activated. CK2 was originally described as a suppressor of FUNDC1 and is associated with FUNDC1 phosphorylation (Chen et al. 2014). The phosphorylation of FUNDC1 directly interacts with LC3B and promotes mitochondrial autophagy (Kuang et al. 2016). Therefore, we speculate that TRF2 plays an important role in FUNDC1‐mediated mitochondrial autophagy by interacting with CSNK2A2. We also overexpressed FUNDC1ser17A in aged cardiomyocytes. After H/Post, mitophagy was activated and the cGAS/STING pathway was inhibited, and H/Post injury was relieved.

This study has certain limitations. Despite the observed downregulation of TRF2 and concomitant telomere shortening during physiological myocardial aging, exogenous overexpression of TRF2 has marginally affected telomere length in aged myocardium. Further investigations using TRF2 knockout mice are therefore needed to fully clarify the regulatory role of TRF2 in telomere homeostasis. Besides, the functional verification of the downstream regulatory factors of TRF2 was conducted in vitro and not in aged mice.

This study aimed to identify targets and pathways that can exert protective effects during I/Post treatment in the aged heart, breaking the vicious cycle of mutual interaction and promotion between myocardial aging, telomere damage, mitochondrial dysfunction, and inflammation caused by I/R. In the future, we hope to translate the newly discovered protective pathways and mechanisms into clinical practice to improve perioperative myocardial injury in older patients.

## Author Contributions

Xuan Zhang, Yiqing Yin conceived the study and designed the experiments. Xuan Zhang, Ji Ma, Ruichen Shu, Yuan Li, Ying Zheng performed the experiments. Xuan Zhang and Ji Ma wrote the manuscript. All authors agreed on its contents before submission.

## Funding

This research was conducted with the support of National Natural Science Foundation of China (82001479 and 82101351).

## Conflicts of Interest

The authors declare no conflicts of interest.

## Supporting information


**Figure S1:** Blocking mtDNA leakage alleviates H/Post injury in aged cardiomyocytes, whereas TRF2 knockdown activates the mtDNA–cGAS–STING signaling axis. (A) qRT‐PCR analysis of cytosolic mtDNA (mt‐Nd1 and mt‐Co1) levels in HL‐1 from the group of H_2_O_2_, H_2_O_2_ + H/Post, H_2_O_2_ + H/Post+EtBr, H_2_O_2_ + H/Post+EtBr+shcon and H_2_O_2_ + H/Post+EtBr+shTRF. (B) Immunoblot analysis of cGAS, STING, Bcl‐2, and Bax protein expression in each group.

## Data Availability

The data that support the findings of this study are available from the corresponding author upon reasonable request.
