# Supplementary material for: TRF2 Recovers Ischemic Postconditioning Cardioprotection in Aged Myocardiocytes by Regulating CSNK2A2 Localization and FUNDC1 Dephosphorylation
Source: Aging Cell. 2026 Aug 17;25(8):e70669. doi: 10.1111/acel.70669 (PMC13479114; doi:10.1111/acel.70669)
Supplement: Supplementary file 1 [file ACEL-25-e70669-s001.docx]

**Supplemental Methods, Figures and Figure Legends**

**Biochemical detection of cytosolic mtDNA release**

Genomic DNA was isolated and purified from the samples using the TIANamp Genomic DNA Kit (Tiangen, China). The concentration and purity of the extracted DNA were determined. The mtDNA target gene was cytochrome coxidase subunit 1 (mt-Co1), mitochondrially encoded NADH dehydrogenase 1(mt-ND1) and the nuclear DNA reference gene was hemoglobin subunit beta (Hbb). The primer sequences (5′→3′) were as follows:

mt-Co1-F: 5′-TGCTAGCCGCAGGCATTAC-3′, R: 5′-GGGTGCCCAAAGAATCAGAAC-3′;

mt‑Nd1‑F: 5′‑CTAGCAGAAACAAACCGGAGG‑3′,mt‑Nd1‑R: 5′‑GGGTGTTGTAAGAGGTGGTTG‑3′;

Hbb-F: 5'-GAAGCGATTCTAGGGAGCAG-3', Hbb-R: 5'-GGAGTGGACAGATCCCCAAG-3' .

The reaction components were as follows: 10 μL of 2× SYBR Green qPCR Master Mix (Takara), 1 μL of forward primer (10 μM), 1 μL of reverse primer (10 μM), 2 μL of template DNA (50 ng/μL), and 6 μL of nuclease-free water. The qRT-PCR was carried out on a real-time PCR instrument (Bio-Rad) using a two-step protocol: pre-denaturation at 95℃ for 3 minutes; 40 cycles of denaturation at 95℃ for 10 seconds and annealing/extension at 60℃ for 30 seconds; and a melting curve analysis (95℃ for 15 seconds, 60℃ for 1 minute, 95℃ for 15 seconds).

**Ethidium bromide (EtBr) induced mtDNA depletion model**

HL-1 cells were treated with 150 μM H_2_O_2_ for 4 h. Cells were cultured in complete medium supplemented with 0.5 μg/mL EtBr (Selleck, Texas, USA) in medium containing uridine (50 μg/mL) and sodium pyruvate (1 mM) for 24 h to establish the mtDNA depletion model. Then, cell transfection was carried out. Subsequently, H/Post was performed.

**Fig. S1 Blocking mtDNA leakage alleviates H/Post injury in aged cardiomyocytes, whereas TRF2 knockdown activates the mtDNA–cGAS–STING signaling axis. A.** qRT-PCR analysis of cytosolic mtDNA (mt-Nd1 and mt-Co1) levels in HL-1 from the group of H_2_O_2_, H_2_O_2_+H/Post, H_2_O_2_+H/Post+EtBr, H_2_O_2_+H/Post+EtBr+shcon and H_2_O_2_+H/Post+EtBr+shTRF. **B.** Immunoblot analysis of cGAS, STING, Bcl-2, and Bax protein expression in each group.
